# Association of Beta-2-Microglobulin With Coronary Heart Disease and All-Cause Mortality in the United States General Population

**DOI:** 10.3389/fcvm.2022.834150

**Published:** 2022-05-13

**Authors:** Yangxi Huang, Yufeng Lin, Xiaobing Zhai, Long Cheng

**Affiliations:** ^1^The Nursing School, Nanjing Medical University, Nanjing, China; ^2^Department of Medicine and Therapeutics, The Chinese University of Hong Kong, Hong Kong, Hong Kong SAR, China; ^3^Child and Adolescent Health, School of Medicine, Wuhan University of Science and Technology, Wuhan, China; ^4^Department of Cardiovascular Medicine, Shanghai Pudong New Area Gongli Hospital, Shanghai, China

**Keywords:** beta-2-microglobulin, coronary heart disease mortality, all-cause mortality, cohort study, risk factor

## Abstract

Few prospective studies explored the association of beta-2-microglobulin (B2M) with coronary heart disease (CHD) mortality. The primary objective of this study was to examine the association of serum B2M with CHD and all-cause mortality. This is a prospective cohort study of a nationally representative sample of 4,885 adults, aged 40–85 years, who participated in the National Health and Nutrition Examination Survey (NHANES III) from 1988 to 1994. The relationships between B2M and CHD and all-cause mortality were estimated using Cox proportional hazards regression models. During a median follow-up of 15.5 years, 845 CHD and 3,388 all-cause deaths occurred among 4,885 participants [2,568 women (55.7%); mean (S.D.) age, 66.4 (12.5) years], respectively. In the unadjusted model, B2M concentration was strongly linearly associated with CHD and all-cause mortality (*p*-trend < 0.001). After adjusting multivariable factors, a positive linear association between B2M and all-cause mortality was still observed (H.R. for Q4 vs. Q1 5.90; 95% CI: 5.31–6.57; *p*-trend < 0.001). In the multivariable adjustment model, B2M was significantly associated with an increased risk of CHD mortality (H.R. for Q4 vs. Q1 2.72; 95% CI: 2.07–3.57; *p*-trend < 0.001). In the stratified analyses, the associations of B2M with CHD and all-cause mortality varied by risk factors, such as age, smoking status, and history of hypertension. The findings suggest a significant relationship between the higher serum B2M concentration and increased risk for CHD and all-cause mortality. Further large-scale follow-up studies are also needed to validate this association.

## Introduction

Coronary heart disease (CHD) is a predominant cause of morbidity and mortality in the United States and worldwide (1), including asymptomatic myocardial ischemia (occult coronary heart disease), angina pectoris, myocardial infarction, and ischemic heart failure (ischemic heart disease) (2). CHD may occur at any age, but the incidence rises progressively with increasing age. However, the specific pathogenesis of CHD is not clear, and it involves multiple processes such as endothelial dysfunction, inflammatory reaction, and lipid deposition hypothesis (3, 4). In recent years, in addition to the traditional risk factors, such as hypertension, hyperlipidemia, smoking, diabetes, obesity, and exercise, an increasing number of scholars are devoting themselves to identify some new risk factors (5–7).

Beta-2-microglobulin (B2M) was first discovered in 1964 in the urine of patients with Wilson’s disease and cadmium poisoning, a histocompatibility complex class I (MHC-I) molecule expressed on almost all nucleated cells (8). Circulating B2M may be a potential biomarker because B2M is associated with inflammatory response and decreased glomerular filtration rate (GFR), and inflammation and impaired GFR in the etiology of vascular diseases (9–11). There is also evidence to suggest that serum B2M concentrations are associated with higher risk of several diseases. In the Atherosclerosis Risk in Communities (ARIC) study, higher serum B2M is associated with increased colorectal cancer risk (12). Additionally, several cohort studies have demonstrated that B2M is associated with an increased risk of chronic kidney disease (13), type 1 diabetes (14), ischemic stroke (15), heart failure (16), cardiovascular disease (17), CHD (15, 17, 18), and so on.

Despite the evidence reporting increased B2M concentrations in CHD patients, few studies have examined serum B2M with CHD and all-cause mortality in general adults. Furthermore, limited evidence of the association between B2M concentrations and CHD risk in the general population is due to their relatively small sample size (17). It is uncertain whether B2M concentrations would be correlated with the prevalence of CHD and all-cause mortality in general adults. It is imperative to understand the impact of serum B2M concentration on CHD and all-cause mortality in the general population. Therefore, the study aimed to determine the association of serum B2M with CHD and all-cause mortality in a nationally representative cohort in the United States.

## Materials and Methods

### Study Design and Population

The National Health and Nutrition Examination Survey (NHANES) is a large-scale, stratified, multistage, ongoing, probability sample survey design of the civilian, non-institutionalized United States population, which was conducted by the National Center for Health Statistics (NCHS) at the Centers for Disease Control and Prevention (CDC) (19, 20). NHANES data are collected from each participant using a household interview, physical examination, and laboratory tests in a mobile examination center. More details of NHANES methods are published elsewhere (21). NHANES has undergone the institutional review board approval. Written and oral informed consent were obtained from participants.

For this analysis, the data were collected from NHANES III (1988 to 1994), because information B2M was available during this period. NHANES III was conducted in two phases over 6 years. Participants completed a household interview, laboratory measurements, and medical examination. Of the 39,695 sample patients aged 2 months and older in the NHANES III, 33,994 (86%) patients were interviewed in participants’ homes. Response rates were high, and 30,818 (78%) of the selected people completed the home interview and the medical examination. Detailed descriptions of NHANES III procedures, interviewing, questionnaires, sample design, analysis guidelines, data collection, and reports of findings have been extensively described previously (20, 22).

Our analytical sample included 4,885 participants, who were above 40 years of age, had no history of heart disease, and had mortality data, including the underlying cause of death and date of death. We excluded those missed data on B2M (*n* = 102), missed data on age (*n* = 475), age < 40 years (*n* = 1938), missed data on demographic variables (*n* = 51), and disease history of CHD (*n* = 356), including congestive heart failure, coronary heart disease, angina pectoris, or heart attack. After these exclusions, a total of 4,885 adults were included in the final analysis (Supplementary eFigure 1).

### Ascertainment of Outcome

The public-use NHANES III Mortality File was used through December 31, 2015, lined by the NCHS to the National Death Index with a probabilistic matching algorithm to determine the mortality status^1^. The National Death Index is a central electronic repository maintained within the NCHS of all deaths in the United States. The NCHS assigned data about the underlying cause of death based on the 10th revision of the International Statistical Classification of Disease (ICD-10) guidelines. We defined deaths from CHD by ICD-10 codes I59-I61 (23). Follow-up of participants continued until death, those who died for reasons other than CHD were also censored at the time of death, or on December 31, 2015, for those who survived. Follow-up time per person was calculated beginning at the NHANES III examination date and ending at the last date known to be alive or censored.

### Exposure Measurement

The B2M was measured in serum using a B2M immunoassay (Siemens Healthcare Diagnostics) on an automated multichannel analyzer. The lower and upper detection limits are 0.72 and 23.0 mg/L, respectively. We analyzed B2M concentrations as categorical by quartiles. B2M concentrations were classified as < 1.800 mg/L, between 1.800 and 2.169 mg/L, between 2.170 and 2.709 mg/L, and ≥ 2.710 mg/L.

### Covariates

Information on age, sex, race/ethnicity, marital status, alcohol intake, smoking status, mean Healthy Eating Index-2010 (HEI-2010), body mass index (BMI) categories, GFR, C-reactive protein, low-density-lipoprotein (LDL) cholesterol, high-density-lipoprotein (HDL) cholesterol, serum globulin, fasting glucose, history of diabetes, history of hypertension, and history of stroke was collected using standardized questionnaires and laboratory protocols during interviews. Race/ethnicity was classified as non-Hispanic white, non-Hispanic black, Mexican American, or other. Marital status was categorized as married, widowed, divorced, and single. Participants were classified as never drinker, moderate drinker, heavy drinker, or missing. Smoking status for each participant was categorized as never smoker, former smoker, and current smoker using two problems, namely, “smoked at least 100 cigarettes in life” and “Do you now smoke cigarettes.” We used the HEI-2010 to evaluate the overall diet quality (range 0–100, with higher scores representing higher quality intakes). BMI was classified as < 25.0 kg/m^2^, 25.0–29.9 kg/m^2^, and ≥ 30.0 kg/m^2^ (24). GFR was calculated from calibrated creatinine, age, sex, and race/ethnicity using the Modification of Diet in Renal Disease Study formula (25). Continuous variables measured in the laboratory include C-reactive protein (mg/dl), LDL cholesterol (mg/dl), HDL cholesterol (mg/dl), serum globulin (g/dl), and fasting glucose (mg/dl). Furthermore, histories of diabetes (yes, no), hypertension (yes, no), and stroke (yes, no) were recorded by self-report.

### Statistical Analysis

We used sample weights, strata, and primary sampling units to explain the complex, multistage, probability sampling in the NHANES. Baseline characteristics of the general population were described using proportions for categorical variables and means (S.E.) for continuous variables. Kaplan–Meier (KM) survival curves were used to evaluate B2M and risk factors associated with CHD and all-cause mortality in the study. Moreover, we applied receiver operating characteristic (ROC) curves to test the area under the curve of the concentration of B2M for predicting the CHD and all-cause mortality. Associations of B2M with CHD and all-cause mortality were identified using Cox proportional hazards models. Hazard ratios (HRs) and 95% confidence intervals (CIs) were adjusted with the following covariates: age (model 1); model 1 plus sex, marital status, and race/ethnicity (model 2); model 2 plus BMI, alcohol intake, and smoking status, C-reactive protein, LDL cholesterol, HDL cholesterol, fasting glucose, serum globulin, GFR, and HEI-2020 score (model 3); and model 3 plus history of diabetes, history of hypertension, and history of stroke (model 4). Besides, analyses were further stratified by age, sex, race/ethnicity, BMI, alcohol intake, smoking status, history of diabetes, history of hypertension, and history of stroke for testing the heterogeneity in the associations of B2M with risk of CHD and all-cause mortality.

Finally, we also conducted sensitivity analyses to evaluate reverse causation bias in the associations by excluding the following: (1) deaths during the first 2 years of follow-up; (2) participants with histories of disease, including diabetes, hypertension, or stroke; (3) participants with GFR < 90 ml/min 1.73 m^2^. Statistical analyses were performed in SAS software version 9.4 and GraphPad Prism 8 software. Two-sided *p*-values < 0.05 were considered statistically significant.

## Results

### Characteristics of the Study Participants

Social demographic characteristics, history of the disease, and continuous biochemical data were recorded and shown in Table 1, according to baseline B2M. Among the 4,885 participants [2,568 women (55.7%); mean (S.D.) age, 66.4 (12.5) years] in the study, during a median 15.5 years of follow-up, 845 CHD and 3,388 all-cause deaths occurred. Compared to participants with the lowest B2M, participants with the highest B2M were more likely to be women, older than 65 years, non-Hispanic white, never drinkers, never smokers, having histories of disease, and higher continuous biochemical variables.

**TABLE 1:** Baseline characteristics according to quartile of B2M.

Characteristic	B2M				
	
	Q1	Q2	Q3	Q4	*P*-value
	<1.800	1.800–2.170	2.170–2.710	>_=_2.710	
Total, N	1,195	1,247	1,209	1,234	
Age, %					<0.001
<65, %	79.76	56.71	33.15	20.2	
≥ 65	20.24	43.29	66.85	79.8	
Sex, %					<0.001
Male	46.92	44.03	45.24	39.68	
Female	53.08	55.97	54.76	60.32	
Race/ethnicity, %^c^					<0.001
Non-Hispanic white	77.03	82.54	86.59	84.6	
Non-Hispanic black	11.56	8.74	6.68	8.95	
Mexican American	4.04	2.55	2.2	1.82	
Other	7.37	6.17	4.53	4.63	
Marital status, %					<0.001
Married	75.66	72.2	62.92	56.82	
Windowed	8.32	15.54	22.39	31.9	
Divorced	10.22	5.9	9.2	5.49	
Single	5.8	6.36	5.49	5.8	
Alcohol intake, %					<0.001
Never drinker	45.48	55.72	60.92	69.3	
Moderate drinker	34.6	26.54	23.19	15.85	
Heavy drinker	19.87	16.89	14.16	12.24	
Missing	0.05	0.85	1.73	2.6	
Smoking, %					<0.001
Never smoker	43.59	43.07	42.44	46.46	
Former smoker	32.73	36.69	40.65	39.46	
Current smoker	23.68	20.24	16.91	14.08	
HEI-2010, mean, SE	64.45 (0.39)	65.80 (0.39)	66.25 (0.38)	65.97 (0.37)	
BMI categories, %					<0.001
<25.0	42.79	35.4	31.09	36.37	
25.0–29.9	37.81	40.24	39.81	33.82	
≥ 30.0	19.4	40.24	39.81	33.82	
GFR, mean, SE	115.85 (0.94)	103.61 (0.78)	94.04 (0.78)	75.29 (0.84)	<0.001
C-reactive protein(mg/dL), mean, SE	0.4 (0.01)	0.45 (0.02)	0.55 (0.02)	0.82 (0.04)	<0.001
LDL-cholesterol (mg/dL), mean, SE	154.43 (2.37)	147.00 (1.43)	154.15 (2.16)	155.01 (2.46)	0.35
HDL-cholesterol (mg/dL), mean, SE	53.67 (0.49)	52.53 (0.46)	50.68 (0.48)	48.94 (0.44)	<0.001
Serum globulin (g/dL), mean, SE	109.59 (1.14)	106.89 (1.25)	106.67 (1.07)	111.79 (1.33)	0.012
Fasting glucose(mg/dL), mean, SE	300.76 (2.29)	313.55 (2.29)	324.2 (2.38)	348.89 (2.88)	<0.001
History of debates, %					<0.001
No	94.19	91.8	91.98	85.34	
Yes	5.81	8.2	8.02	14.66	
History of hypertension, %					
No	74.31	67.14	58.17	44.62	<0.001
Yes	25.69	32.86	41.83	55.38	
History of stroke, %					<0.001
No	98.76	97.17	95.7	90.04	
Yes	1.24	2.83	4.3	9.6	

*Values are weighted mean ± S.E. for continuous variables or weighted% for categorical variables.*

### Association of B2M With CHD and All-Cause Mortality

**FIGURE 1:**
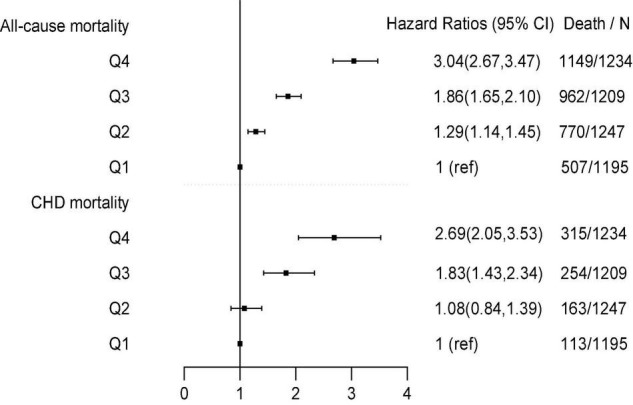
Associations of B2M with CHD and all-cause mortality.

Table 2 and Figure 1 show Cox proportional HRs and 95% CIs for all-cause and CHD mortality by quartiles of B2M. B2M was strongly associated with all-cause mortality (HR for Q4 vs. Q1 5.90; 95% CI: 5.31–6.57; *p*-trend < 0.001) in the unadjusted model. After further adjustment for sex, race/ethnicity, marital status, BMI, alcohol, smoking, GFR, C-reactive protein, LDL cholesterol, HDL cholesterol, serum globulin, fasting glucose, and HEI-2010, B2M was still significantly associated with all-cause mortality in model 2. In multivariable model 3, a positive linear association between B2M and all-cause mortality was observed (HR for Q4 vs. Q1 3.04; 95% CI: 2.67–3.47; *p*-trend < 0.001). A similar association was observed for CHD mortality. In the unadjusted model, B2M was strongly correlated with CHD mortality (HR for Q4 vs. Q1 6.75; 95% CI: 5.42–8.41; *p*-trend < 0.001). In multivariable adjustment model 3, we observed a positive linear association between B2M and CHD mortality (HR for Q4 vs. Q1 2.69; 95% CI: 2.05–3.53; *p*-trend < 0.001). In addition, a dose-response relationship between B2M and CHD and all-cause mortality was observed, and deaths increased with the increase in B2M concentration.

**TABLE 2:** The association of B2M with CHD and all-cause mortality.

	B2M				
	
	Q1 <1.800	Q2 1.800–2.169	Q3 2.170–2.709	Q4 >_=_2.710	*P trend*
**All-cause mortality**					
Deaths, No. (%)	507 (34.5)	770 (55.7)	962 (77.4)	1,149 (91.0)	
Unadjusted	1.00 (Reference)	1.76 (1.57, 1.97)	2.94 (2.64, 3.28)	5.90 (5.31, 6.57)	<0.001
Model1	1.00 (Reference)	1.28 (1.14, 1.44)	1.84 (1.64, 2.06)	3.21 (2.87, 3.60)	<0.001
Model2	1.00 (Reference)	1.30 (1.15, 1.46)	1.87 (1.66, 2.10)	3.19 (2.80, 3.63)	<0.001
Model3	1.00 (Reference)	1.29 (1.14, 1.45)	1.86 (1.65, 2.10)	3.04 (2.67, 3.47)	<0.001
**CHD mortality**					
Deaths, No. (%)^a^	113 (7.3)	163 (11.9)	254 (20.6)	315 (23.4)	
Unadjusted	1.00 (Reference)	1.64 (1.29, 2.09)	3.38 (2.70,4.23)	6.75 (5.42, 8.41)	<0.001
Model1	1.00 (Reference)	1.14 (0.89, 1.45)	1.94 (1.53, 2.45)	3.36 (2.66, 4.25)	<0.001
Model2	1.00 (Reference)	1.09 (0.85, 1.40)	1.82 (1.43, 2.33)	2.86 (2.19, 3.74)	<0.001
Model3	1.00 (Reference)	1.08 (0.84, 1.39)	1.83 (1.43, 2.34)	2.69 (2.05, 3.53)	<0.001

*Values are n or hazard ratio (95% confidence interval).*

*Unadjusted model: B2M.*

*Model 1: unadjusted model + age, sex, race/ethnicity, and marital status.*

*Model 2: model 2 + BMI, alcohol, smoking, GFR, C-reactive protein, LDL cholesterol, HDL cholesterol, serum globulin, fasting glucose, and HEI-2010.*

*Model 3: model 3 + history of diabetes mellitus, history of hypertension, and history of stroke.*

*CHD = coronary heart disease.*

As shown in Supplementary eFigures 1, 2, participants with the highest B2M relative to their lowest B2M had much steeper declines in survival over more than 20 years of follow-up. Among individuals with the highest B2M at baseline, about 90% of individuals for all-cause mortality and 50% of individuals for CHD mortality in the lowest B2M group died after 25 years of follow-up. Conversely, among individuals with the lowest B2M, only about 50% of individuals for all-cause mortality and 15% of individuals for CHD mortality in the highest B2M group had died.

The ROC curves for all-cause mortality (Supplementary eFigure 2A) revealed that B2M, with an AUC of 0.76 (95% CI: 0.75–0.78), significantly outperformed the participants’ serial biochemical data measures and other risk factors. The next highest performing measures were plasma fibrinogen, with an AUC of 0.60 (95% CI: 0.59–0.62); serum glucose, with an AUC of 0.58 (95% CI: 0.56–0.59); and C-reactive protein, with an AUC of 0.55 (95% CI: 0.53–0.56). Three measures, namely, LDL cholesterol, HDL cholesterol, and BMI, had AUCs between 0.45 and 0.51, and 2 measures, namely, HDL cholesterol and BMI, had AUCs less than 0.50. In addition, ROC curves for CHD mortality revealed B2M are shown in Supplementary eFigure 2B.

### Stratification Analysis

**FIGURE 2:**
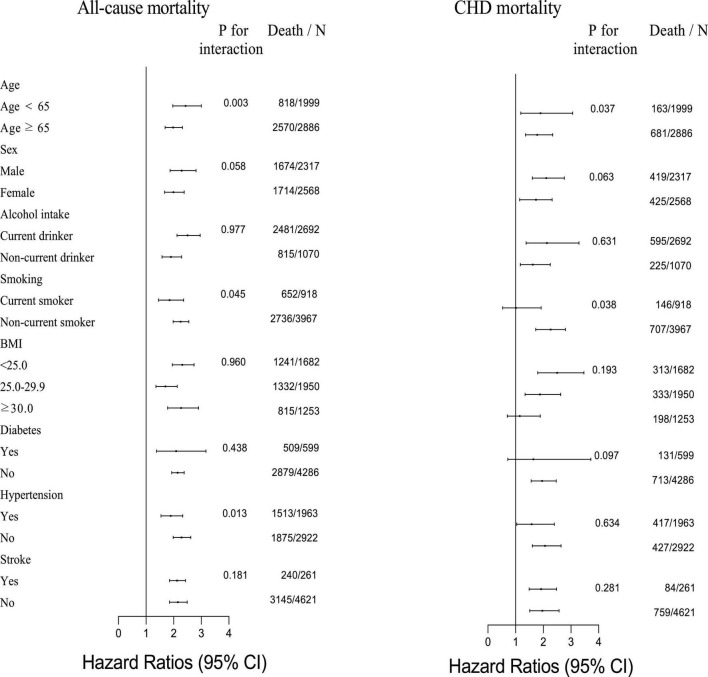
Subgroup analysis of the association of B2M with CHD and all-cause mortality.

The results of the stratification analysis of CHD and all-cause mortality are shown in Figure 2, considering age, sex, BMI, alcohol intake, smoking status, history of diabetes, history of hypertension, and history of stroke. There was a significant difference in the association between B2M and all-cause mortality for the stratification by age, smoking status, and history of hypertension. The significantly strongly association was observed in participants with age < 65 years, non-current smoking, and non-hypertension; and their HRs (95% CIs) were 2.43 (1.97–3.00), 2.25 (1.98–2.55), and 2.28 (1.98–2.62), respectively. Similarly, the associations between B2M and CHD mortality indeed exist discrepancy by age and smoking status. The HRs (95% CIs) were 1.90 (1.19–3.06) for participants with age < 65 years and 2.22 (1.75–2.82) for participants with non-current smoking.

### Sensitivity Analysis

Excluding deaths (all-cause mortality *n* = 221 or CHD mortality *n* = 72) during the first 2 years of follow-up showed that the association of the proportion of B2M with CHD and all-cause mortality was similar to our main findings (Supplementary eTable 1). In addition, the results (Supplementary eTable 2) showed that associations of B2M with CHD and all-cause mortality were similar to our primary analysis when participants with histories of disease (diabetes, hypertension, and stroke, *n* = 2316) were excluded. Finally, to test the influence of GFR, we excluded participants with GFR < 90 ml/min⋅1.73 m^2^. However, the association of B2M with CHD and all-cause mortality did not change significantly (Supplementary eTable 3).

## Discussion

In this nationally representative cohort of United States adults aged 20 years and older, we found that a high B2M concentration was positively associated with an increased risk of CHD and all-cause mortality. The association between B2M and all-cause mortality was modified by age, smoking status, and history of hypertension. Records of age and smoking status modified the association with CHD mortality.

This study is one of the first studies to explore the association of B2M with CHD and all-cause mortality, which is consistent with previous studies on the relationship between serum B2M and all-cause mortality. Serum B2M concentration was positively correlated with the general population (26) and patients with the chronic obstructive pulmonary disease (27), type 2 diabetes (28), and acute coronary syndrome (29). A prospective cohort study with 708 all-deaths among 6,445 general population demonstrated that after adjusting mortality risk factors, compared with the middle quintile, the highest sub-quintiles for B2M (Q5c: HR, 2.58; 95%CI: 1.96–3.41) were associated with all-cause mortality risk (17). A meta-analysis of 16 studies with 6,165 all-cause deaths among 30,988 subjects showed that the individuals in the highest B2M distribution had a higher risk of all-cause mortality compared with the lowest B2M distribution (RR: 2.51, 95% CI: 1.94–3.26; I2 = 83.7%, *p*-trend < 0.001) (18). The significant association between B2M and all-cause mortality is plausible if we consider a positive association between serum B2M concentration and major causes of death, such as cancer (12, 30), CVD (16, 18), and other chronic and infectious diseases (31, 32), due to pro-angiogenic, pro-tumorigenic, driving innate pro-inflammatory cytokines [e.g., TNF-α, interleukin (IL)-1, IL-6, and IL-8] and growth-promoting factors (e.g., vascular endothelial growth factor, epidermal growth factor receptor, fatty acid synthase, insulin, and insulin-like growth factor 1 receptor), and epithelial-mesenchymal transition (12).

The CHD is the leading global cause of mortality, so there is great interest in discovering novel biomarkers that distinguish individuals at a higher risk of CHD. Circulating B2M may be a potential biomarker given the associations of elevated B2M with inflammatory responses and declining GFR, together with the involvement of inflammation and impaired GFR in the pathogenesis of vascular disease. Recent epidemiology studies showed a higher B2M concentration associated with higher CHD risk (16, 26). A Framingham Heart Study (16) identified B2M that can predict a higher risk of CHD mortality: HR = 1.24, 95CI%: 1.10–1.40. The ARIC study (26) with a total of 1,279 CHD events among 9,988 subjects indicated that B2M concentrations were more strongly associated with CHD risks. Moreover, a meta-analysis involving 30,988 participants and 5,391 CVD events showed that B2M concentration was positively related to CHD incidence rate; pooled adjusted RRs comparing the highest versus the lowest third of the distribution of B2M was 1.64 (95%CI: 1.14–2.34) (18). Although several previous studies have evaluated the association of B2M with incidence rate and mortality of CHD (16, 18, 26), it is currently inconsistent with the relationship between serum B2M concentration and CHD mortality. For example, a prospective cohort study identified that only the B2M sub-quintiles 5c were associated with CHD mortality (HR = 2.15, 95CI%: 1.30–3.56) (17). In addition, in our prospective cohort study, we primarily found positive associations between B2M quartile 3 (HR = 1.83, 95%CI: 1.43–2.34) and quartile 4 (HR = 2.72, 95%CI: 2.07–3.57) and CHD mortality, supporting B2M as a new predictor of CHD.

To further evaluate the relationship between B2M and CHD and all-cause mortality, we performed the stratified analyses by age, sex, BMI, smoking, alcohol intake statuses, history of stroke, hypertension, and diabetes separately. Results showed significant modifications in the association between B2M and all-cause mortality, for age, smoking status, and the history of hypertension. On the contrary, the association between B2M and CHD mortality varied by age and smoking status.

Both clinical and experimental studies accumulated so far suggest a causal relationship between hypertension and a series of functional disorders of the body (33, 34). Mechanisms for dysfunction after hypertension are very complicated, including inflammatory activation (35), neurogenic abnormality (36), endothelial dysfunction caused by circulating asymmetric dimethylarginine (37), and oxidized LDL (38), and may be involved in the association between hypertension and the risk of all-cause mortality. However, considering the interaction effect between B2M and hypertension, assessing the association between B2M and all-cause risk may be limited by the effect of hypertension on all-cause mortality. Smoking is one of the cornerstones of CHD and all-cause mortality, a major cause of health problems, and determines more than 30% of CHD mortality (39, 40). Smoking causes oxidation process, which has a negative impact on platelet function, fibrinolysis, inflammation, and vasomotor function. All these pro-atherosclerotic effects doubled the risk of fatal events in smokers within 10 years compared with non-smokers (39), which confer increased risk of CHD and all-cause mortality regardless of the serum B2M concentration, and may mask the predictive role of B2M on mortality in smokers. Similarly, age can also hide the association of B2M concentration, due to the additional effect of age on mortality.

The biological mechanisms underlying the relationship between the serum B2M concentration and CHD and all-cause mortality are not well established. Emerging evidence supports the hypothesis that B2M plays a role associated with inflammatory changes. CHD results from a build-up of cholesterol accumulation along the arterial walls. However, CHD is a complex disease process whose pathogenic basis extends far beyond intimal infiltration of cholesterol. Numerous clinical and experimental evidence show that inflammation is pivotal in the development and progression of atherosclerosis (41). Uncontrolled inflammation plays a central role in making a carotid plaque unstable and vulnerable to rupture or erosion, which causes thrombosis, myocardial infarction, and sudden death. Also, numerous studies demonstrated that inflammation is related to a higher risk of all-cause mortality (42–44). Some have suggested that B 2M may have a direct pathogenic role in immune response and inflammation or may serve as an early biomarker of increased cell turnover (17, 31, 45, 46). Its role as an inflammation marker is corroborated by studies reporting positive associations of B2M with CVD (14), diabetes (17), and mortality from those diseases and all causes. Thus, inflammation might be a potential mechanism linking B2M and CHD and all-cause mortality.

The strengths of our study include the use of a nationally complex, comprehensive, multistage, and representative sample of United States adults, whose large sample size and extended follow-up allowed adequate statistical power to examine the associations of B2M with CHD and all-cause mortality. Moreover, we excluded people with chronic diseases from sensitivity analysis because their prevalent health conditions might affect the associations of B2M with CHD and all-cause mortality. However, there was no difference in associations of B2M with CHD and all-cause mortality compared with the main findings of this study.

This study has several limitations. First, the serum B2M concentration was measured only at baseline, and participants’ serum B2M may have changed over time. Second, residual confounding may still exist, although a number of covariates were adjusted. Third, mortality outcomes were determined through linkage to the National Death Index with a probabilistic matching algorithm to determine the mortality status that may result in misclassification. Finally, caution is needed to generalize our results to non-US populations due to different nationalities, living environments, and chronic disease epidemiology.

## Conclusion

Based on a representative sample of American adults, a high B2M concentration was significantly associated with an increased risk of CHD mortality. Measurement of B2M may provide a responsive biomarker for early indication of CHD and therapy guidance. However, future studies are also required to validate our findings and determine their clinical utility, with the ultimate goal finding more targeted ways for CHD prevention.

## Data Availability Statement

The datasets presented in this study can be found in online repositories. The names of the repository/repositories and accession number(s) can be found in the article/Supplementary Material.

## Ethics Statement

The studies involving human participants were reviewed and approved by Ethics Committee of Nanjing Medical University. The patients/participants provided their written informed consent to participate in this study.

## Author Contributions

YH, XZ, and LC contributed to the conception, design, and analysis of the work. YH and LC drafted the work. LC, YL, and XZ revised the manuscript. YH, YL, XZ, and CL approved the version to be published. All authors contributed to the article and approved the submitted version.

## Conflict of Interest

The authors declare that the research was conducted in the absence of any commercial or financial relationships that could be construed as a potential conflict of interest.

## Publisher’s Note

All claims expressed in this article are solely those of the authors and do not necessarily represent those of their affiliated organizations, or those of the publisher, the editors and the reviewers. Any product that may be evaluated in this article, or claim that may be made by its manufacturer, is not guaranteed or endorsed by the publisher.

## Footnotes

1
https://www.cdc.gov/nchs/data-linkage/mortality-public.htm
