# Supplementary material for: Association of Beta-2-Microglobulin With Coronary Heart Disease and All-Cause Mortality in the United States General Population
Source: Front Cardiovasc Med. 2022 May 13;9:834150. doi: 10.3389/fcvm.2022.834150 (PMC9136227; doi:10.3389/fcvm.2022.834150)
Supplement: Supplementary file 1 [file Data_Sheet_1.pdf]

eTable 1 Sensitivity Analysis for CHD and All-cause Mortality Associated with B2M, excluding deaths during the first 2 years of follow-up (all-cause mortality n=221 or CHD mortality n=72)

	B2M				
	Q1<br>< 1.800	Q2<br>1.800-2.169	Q3<br>2.170-2.709	Q4<br>≧2.710	*P trend*
All-cause mortality					
Deaths, No. (%)	489（33.7）	750（55.3）	918（76.8）	1010（90.0）	
Unadjusted	1.00 [Reference]	1.79(1.59,2.00)	2.97(2.66,3.32)	5.72(5.12,6.38	<0.001
Model1	1.00 [Reference]	1.30(1.16, 1.47)	1.84(1.64,2.07)	3.09(2.75,3.48)	<0.001
Model2	1.00 [Reference]	1.32(1.17,1.48)	1.87(1.65,2.11)	3.07(2.68,3.51)	<0.001
Model3	1.00 [Reference]	1.29(1.15,1.45)	1.84(1.63,2.08)	2.88(2.51,3.30)	<0.001
CHD mortality					
Deaths, No. (%) [a]	113（7.1）	163（11.6）	254（20.3）	315（23.2）	
Unadjusted	1.00 [Reference]	1.62(1.27,2.08)	3.40(2.71,4.27)	6.45(5.14,8.10)	<0.001
Model1	1.00 [Reference]	1.10(0.86,1.42)	1.90(1.50,2.42)	3.14(2.46,4.00)	<0.001
Model2	1.00 [Reference]	1.06(0.82,1.37)	1.78(1.38,2.29)	2.69(2.04,3.55)	<0.001
Model3	1.00 [Reference]	1.05(0.81,1.36)	1.78(1.38,2.30)	2.52(1.90,3.35)	<0.001

Values are n or hazard ratio (95% confidence interval).

Unadjusted model: B2M.

Model 1: unadjusted model +age, sex, race/ethnicity, and marital status.

Model 2: model 1 + BMI, alcohol, smoking, GFR, C-reactive protein, LDL-cholesterol, HDL-cholesterol, Serum globulin, Fasting glucose, and HEI-2010.

Model 3: model 2 +history of diabetes mellitus, history of hypertension, and history of stroke.

CHD = coronary heart disease

eTable 2 Sensitivity Analysis for CHD and All-cause Mortality Associated with B2M, excluding participants with history of disease (diabetes, hypertension, and stroke, n=2316)

	B2M				
	Q1<br>< 1.800	Q2<br>1.800-2.169	Q3<br>2.170-2.709	Q4<br>≧2.710	*P trend*
All-cause mortality					
Deaths, No. (%)	272（29.4）	414（49.0）	475（73.7）	420（86.4）	
Unadjusted	1.00 [Reference]	1.94(1.67,2.26)	3.44(2.93,4.00)	6.72(5.75,7.85)	<0.001
Model1	1.00 [Reference]	1.21(0.85,1.73)	1.93(1.36,2.72)	2.70(1.87,3.91)	<0.001
Model2	1.00 [Reference]	1.28(0.89,1.84)	2.15(1.49,3.12)	3.19(2.10,4.83)	<0.001
CHD mortality					
Deaths, No. (%) [a]	54（4.9）	84（10.7）	116（17.9）	93（16.7）	
Unadjusted	1.00 [Reference]	1.98(1.40,2.79)	4.11(2.97,5.70)	6.92(4.91,9.75)	<0.001
Model1	1.00 [Reference]	1.23(0.86,1.75)	1.94(1.37,2.74)	2.74(1.89,3.96)	<0.001
Model2	1.00 [Reference]	1.26(0.88,1.80)	2.08(1.44,3.01)	3.10(2.05,4.68)	<0.001

Values are n or hazard ratio (95% confidence interval).

Unadjusted model: B2M.

Model 1: unadjusted model +age, sex, race/ethnicity, and marital status.

Model 2: model 1 + BMI, alcohol, smoking, GFR, C-reactive protein, LDL-cholesterol, HDL-cholesterol, Serum globulin, Fasting glucose, and HEI-2010.

CHD = coronary heart diseas

eTable 3 Sensitivity Analysis for CHD and All-cause Mortality Associated with B2M, excluding eGFR < 60 mL·min^{-1}·1.73 m^{-2} (n=2129)

	B2M				
	Q1<br>< 1.800	Q2<br>1.800-2.169	Q3<br>2.170-2.709	Q4<br>≧2.710	*P trend*
All-cause mortality					
Deaths, No. (%)	407（33.5）	523（58.2）	504（75.3）	268（84.4）	
Unadjusted	1.00 [Reference]	1.76(1.57,1.97)	2.94(2.64,3.28)	5.90(5.31,6.57)	<0.001
Model1	1.00 [Reference]	1.28(1.14,1.44)	1.84(1.64,2.06)	3.21(2.87,3.60)	<0.001
Model2	1.00 [Reference]	1.29(1.15,1.45)	1.85(1.65,2.08)	3.14(2.79,3.53)	<0.001
Model3	1.00 [Reference]	1.28(1.14,1.43)	1.84(1.64,2.06)	2.97(2.64,3.35)	<0.001
CHD mortality					
Deaths, No. (%) [a]	93（7.1）	111（13.0）	132（18.4）	60（16.8）	
Unadjusted	1.00 [Reference]	1.63(1.24,2.15)	3.19(2.44,4.17)	3.97(2.86,5.52)	<0.001
Model1	1.00 [Reference]	1.11(0.84,1.48)	1.97(1.48,2.63)	2.36(1.67,3.35)	<0.001
Model2	1.00 [Reference]	1.11(0.83,1.48)	1.96(1.47,2.62)	2.14(1.49,3.07)	<0.001
Model3	1.00 [Reference]	1.09(0.81,1.45)	1.94(1.45,2.61)	1.98(1.37,2.87)	<0.001

Values are n or hazard ratio (95% confidence interval).

Unadjusted model: B2M.

Model 1: unadjusted model +age, sex, race/ethnicity, and marital status.

Model 2: model 1 + BMI, alcohol, smoking, C-reactive protein, LDL-cholesterol, HDL-cholesterol, Serum globulin, Fasting glucose, and HEI-2010.

Model 3: model 2 +history of diabetes mellitus, history of hypertension, and history of stroke.

CHD = coronary heart disease

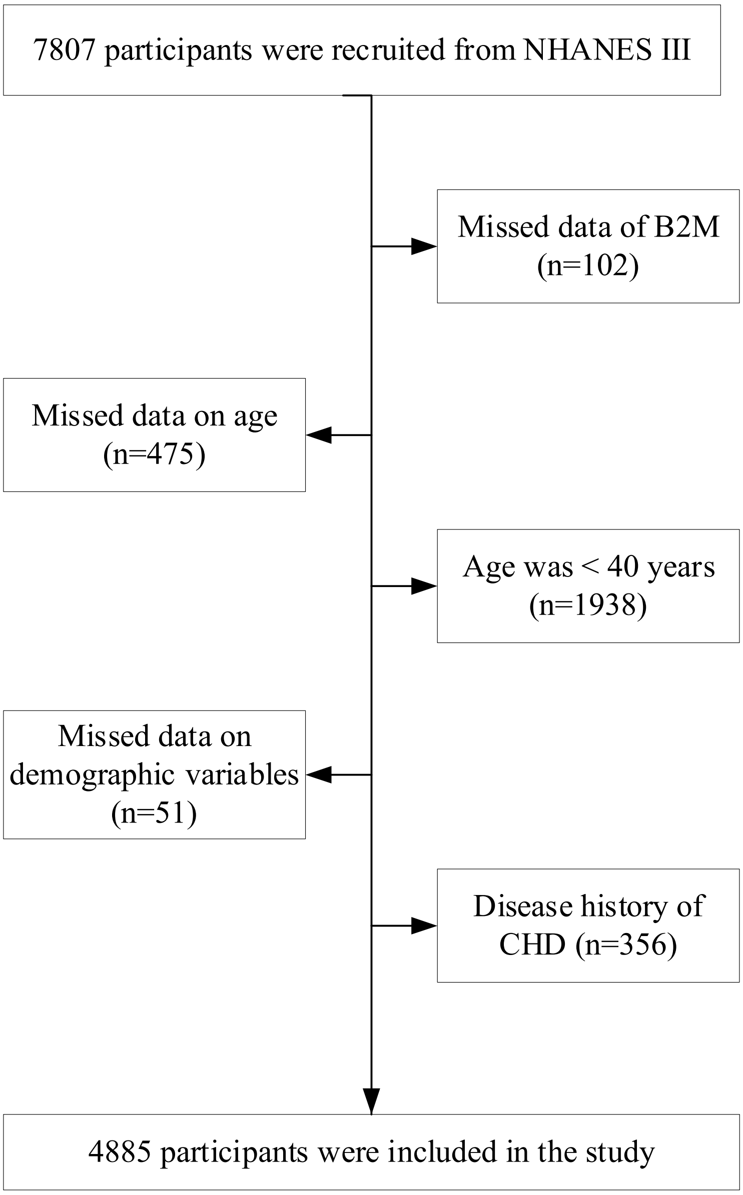

eFigure 1 Sample selection process

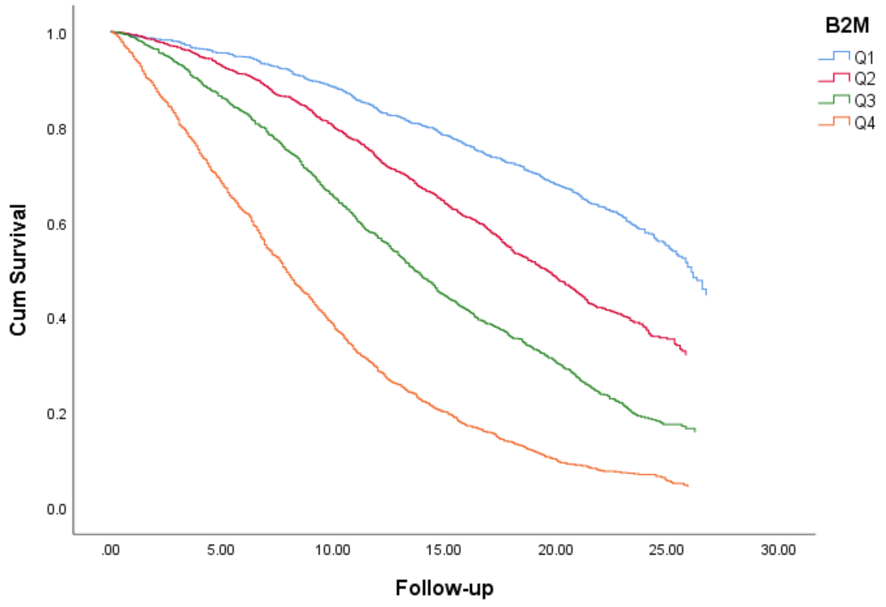

eFigure 2 Kaplan–Meier curves for all-cause mortality

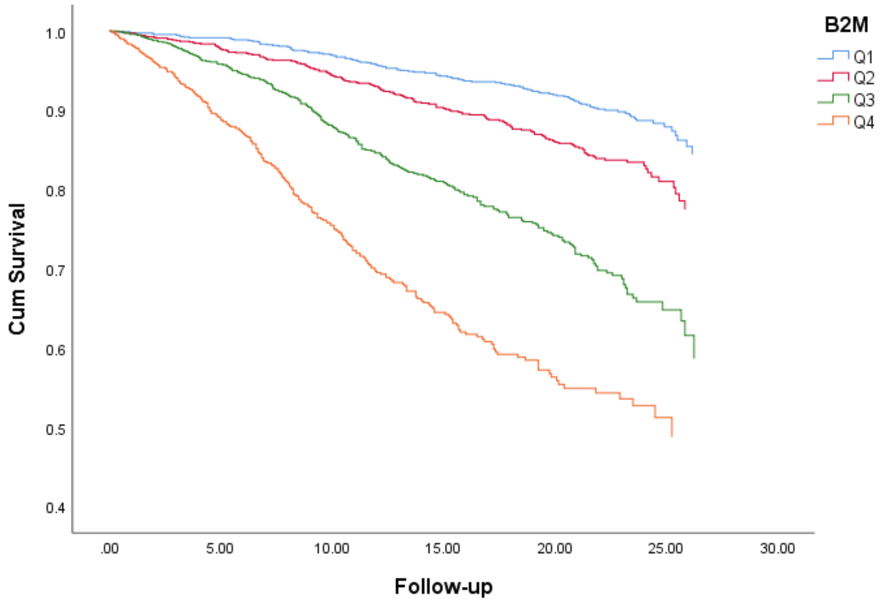

eFigure 3 Kaplan–Meier curves for CHD mortality.

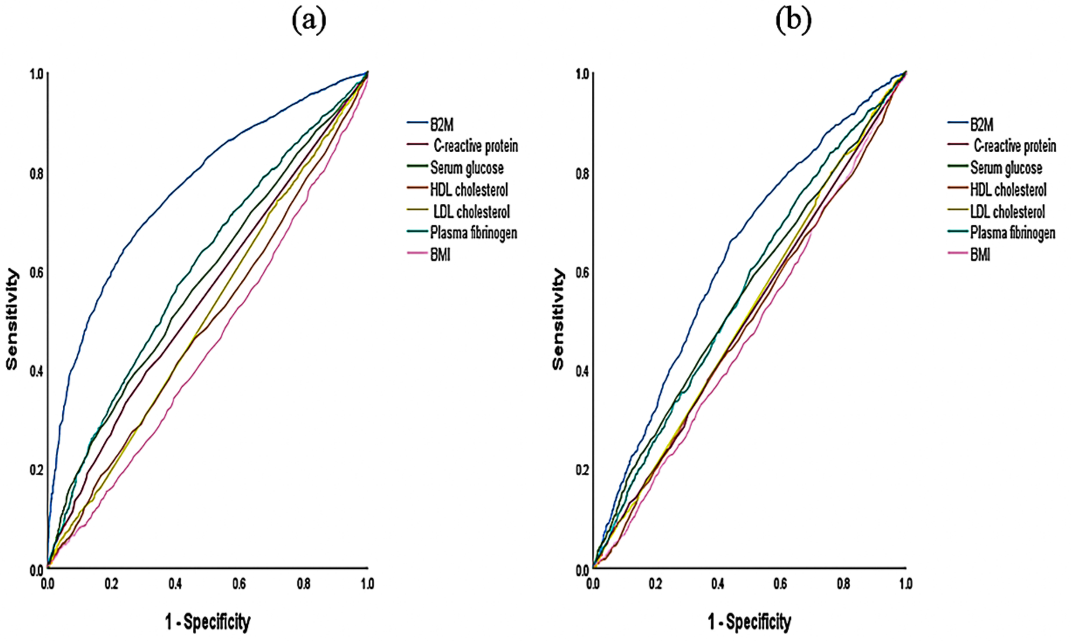

	AUC	Bonferroni P-value	95% CI
All-cause mortality			
B2M	0.76	<0.001	(0.75,0.78)
Plasma fibrinogen	0.60	<0.001	(0.59,0.62)
Serum glucose	0.58	<0.001	(0.56,0.59)
C-reactive protein	0.55	<0.001	(0.53,0.56)
LDL cholesterol	0.51	0.502	(0.49,0.52)
HDL cholesterol	0.49	0.239	(0.47,0.51)
BMI	0.45	<0.001	(0.43,0.47)
CHD			
B2M	0.628	<0.001	(0.61,0.65)
Plasma fibrinogen	0.559	<0.001	(0.54,0.58)
Serum glucose	0.551	<0.001	(0.53,0.57)
C-reactive protein	0.505	0.632	(0.48,0.53)
LDL cholesterol	0.514	0.199	(0.49,0.54)
HDL cholesterol	0.492	0.441	(0.47,0.51)
BMI	0.478	0.047	(0.46,0.50)

eFigure 4 Receiver operating characteristic curves for mortality.
AUC = area under the curve; BMI = body mass index.
